# Assessment of sedentary behaviors and transport-related activities by questionnaire: a validation study

**DOI:** 10.1186/s12889-016-3412-3

**Published:** 2016-08-09

**Authors:** Keitly Mensah, Aurélia Maire, Jean-Michel Oppert, Julien Dugas, Hélène Charreire, Christiane Weber, Chantal Simon, Julie-Anne Nazare, ACTI-Cités consortium, Thomas Bastian, Stéphane Blanc, Hélène Charreire, Julien Dugas, Christophe Enaux, Thierry Feuillet, Franck Hess, Mehdi Menai, Julie-Anne Nazare, Jean-Michel Oppert, Camille Perchoux, Paul Salze, Chantal Simon, Christiane Weber

**Affiliations:** 1 CRNH Rhône-Alpes/CENS, Hospices Civils de Lyon, Lyon, France; 2 Univ-Lyon, CarMeN laboratory, Inserm U1060, INRA U1397, Université Claude Bernard Lyon 1, INSA Lyon, Charles Merieux Medical School, Fr-69600, Oullins, France, Lyon, France; 3 Université Paris 13, Sorbonne Paris Cité - EREN (Equipe de Recherche en Epidémiologie Nutritionnelle), U1153 Inserm, Inra, Cnam, Centre de Recherche en Epidémiologie et, Biostatistiques, CRNH IdF, Bobigny, France; 4 Department of Nutrition Pitié-Salpêtrière Hospital (AP-HP), Université Pierre et Marie Curie-Paris 6, Paris, France; 5 Université Paris-Est, Lab-Urba, Créteil, France; 6 Laboratoire Image, Ville et Environnement, Université de Strasbourg, Strasbourg, France; 7 Service d’Endocrinologie, Diabète, Nutrition Centre Hospitalier Lyon Sud, 165 Chemin du Grand Revoyet, F69310 Pierre-Bénite, France

**Keywords:** Sedentary behavior, Sitting, Active transport, Travel, Logbook, Accelerometry, Context, Questionnaire

## Abstract

**Background:**

Comprehensive assessment of sedentary behavior (SB) and physical activity (PA), including transport-related activities (TRA), is required to design innovative PA promotion strategies. There are few validated instruments that simultaneously assess the different components of human movement according to their context of practice (e.g. work, transport, leisure). We examined test-retest reliability and validity of the Sedentary, Transportation and Activity Questionnaire (STAQ), a newly developed questionnaire dedicated to assessing context-specific SB, TRA and PA.

**Methods:**

Ninety six subjects (51 women) kept a contextualized activity-logbook and wore a hip accelerometer (Actigraph GT3X + ^TM^) for a 7-day or 14-day period, at the end of which they completed the STAQ. Activity-energy expenditure was measured in a subgroup of 45 subjects using the double labeled water (DLW) method. Test-retest reliability was assessed using intra-class-coefficients (ICC) in a subgroup of 32 subjects who filled the questionnaire twice one month apart. Accelerometry was annotated using the logbook to obtain total and context-specific objective estimates of SB. Spearman correlations, Bland-Altman plots and ICC were used to analyze validity with logbook, accelerometry and DLW data validity criteria.

**Results:**

Test-retest reliability was fair for total sitting time (ICC = 0.52), good to excellent for work sitting time (ICC = 0.71), transport-related walking (ICC = 0.61) and car use (ICC = 0.67), and leisure screen-related SB (ICC = 0.64-0.79), but poor for total sitting time during leisure and transport-related contexts. For validity, compared to accelerometry, significant correlations were found for STAQ estimates of total (*r* = 0.54) and context-specific sitting times with stronger correlations for work sitting time (*r* = 0.88), and screen times (TV/DVD viewing: *r* = 0.46; other screens: *r* = 0.42) than for transport (*r* = 0.35) or leisure-related sitting-times (*r* = 0.19). Compared to contextualized logbook, STAQ estimates of TRA was higher for car (*r* = 0.65) than for active transport (*r* = 0.41). The questionnaire generally overestimated work- and leisure-related SB and sitting times, while it underestimated total and transport-related sitting times.

**Conclusions:**

The STAQ showed acceptable reliability and a good ranking validity for assessment of context-specific SB and TRA. This instrument appears as a useful tool to study SB, TRA and PA in context in adults.

**Electronic supplementary material:**

The online version of this article (doi:10.1186/s12889-016-3412-3) contains supplementary material, which is available to authorized users.

## Background

There is increasing evidence documenting relationships between sedentary behaviors (SB) and the risk of chronic diseases such as type 2 diabetes, cardiovascular disease, independent of the level of moderate-to-vigorous physical activity (PA) [1–3]. As for cancers, associations with SB have been reported in some studies, although the evidence is less consistent [4, 5]. SB are defined as a class of waking behaviors, performed when in a sitting or reclining position, and associated with low levels of energy expenditure [6]. Time spent watching TV (or screen viewing) and total sitting time are typical, although very global SB indicators used in epidemiological studies [3]. Several recent studies have also suggested beneficial associations between active transportation and cardiometabolic risk [7–9], pointing out the critical role of transport-related activities (TRA) in movement behavior. In this context, an integrated and comprehensive approach of SB and PA to better measure, understand and promote human movement as a whole, is required to implement successful interventions for the promotion of a healthy lifestyle [10–13]. Nevertheless, the circumstances and contexts in which activities are performed, beyond their duration, frequency and intensity, are still barely addressed [6, 14]. For this purpose, there is a need of measurement tools that accurately, as well as concurrently, assess SB and TRA according to the context (e.g. work, transport, domestic life, leisure,) in which daily activities are performed.

SB, as much as PA, can be measured using objective or subjective methods [14]. Besides estimates of activity energy expenditure (AEE), activity monitors allow objective assessment of total time spent sedentary, with both advantages and limitations linked to how they record sedentary and sitting time [15]. Usually accelerometry-based monitors do not directly distinguish specific posture such as lying or sitting/reclining from standing but use a specific counts threshold (e.g. less than 100 or 150 counts/min) to identify sedentary activities that are associated with low energy expenditure. More recently, inclinometry has been used to objectively assess posture and thus more specifically record time spent sitting. However if inclinometry is a valid tool to distinguish lying/sitting from standing when the device is thigh-mounted (which is poorly convenient for long-term recordings), misclassification of these postures is rather high when the device is worn at the waist or the hip [16, 17]. In any case, when it comes to specifically identifying the context where activities take place, subjective methods such as questionnaires or logbooks remain unique tools [14]. In logbooks, individuals are instructed to prospectively record the time spent during the day, typically by 15-to-30 min blocks, in a broad range of activities and potentially their context of practice. However, logbooks, which have been recently used concurrently with accelerometry or inclinometry to produce objective measures of context-specific SB [18], appear more time-consuming and require more personal investment from subjects than questionnaires. Questionnaires remain thus a method of choice for large-scale epidemiological studies since they are easily implemented at a low cost.

While emerging evidence highlights the importance of taking into account the context of activities in regards to its influence on both behavior determinants and health impact [3, 19, 20], only few works, mainly focusing SB, have incorporated this context-dimension [17, 18, 21, 22]. To our knowledge, there is no published questionnaires that allow simultaneously assessing context-specific SB and PA, including TRA. The design and the validation of a questionnaire addressing this gap was one objective of the ACTI-Cités project, a multidisciplinary project aiming to better understand the relationships of transport behaviors with the environmental characteristics in a large sample of French adults, the “Nutrinet” cohort [23]. For this purpose, based on leisure and occupational PA questions from the Recent Physical Activity Questionnaire (RPAQ) [24], we added questions specifically addressing context-specific SB and TRA to develop a dedicated questionnaire, the Sedentary, Transportation and Activity Questionnaire (STAQ).

The main objective of the present study was to examine the test-retest reliability and the validity of the STAQ items assessing total and context-specific SB and TRA. Validity criterion measures were 1) total and context-specific sedentary time derived from accelerometry (Actigraph GT3X + ^TM^) annotated using contextualized logbook, 2) total and context-specific TRA derived from contextualized logbook. A secondary objective was to compare the questionnaire AEE-estimate with its measure by the double-labelled water (DLW) reference method.

## Methods

### Development of the sedentary, transportation and activity questionnaire

The Sedentary, Transportation and Activity Questionnaire (STAQ) is a self-administered questionnaire collecting past-month activity data in a disaggregated way, so that information may be summarized according to different contexts: domestic life (at the exclusion of housework activities), work, commuting and transport-related, and leisure. The STAQ is based on the French version of the RPAQ that has been shown to be a valid instrument for ranking individuals according to their AEE and time spent at vigorous-intensity PA, but to produce a weaker assessment of time spent at light-to-moderate-intensity PA [24, 25], and in which questions have been modified or added to more specifically address different context-specific SB and TRA. The STAQ is divided in 4 sections addressing physical activity in different settings (Home; Activity at work/studying; Transport and Recreation/Leisure) and an additional section with more synthetic questions, including questions on subject’s physical activity perception.

To assess the passive and active transport modes that represent a high proportion of time spent in respectively SB and light-to-moderate PA, detailed questions were asked on time spent in different transport modes (car or motorized vehicle, public transport, walking, cycling, other active transport) assessing the past-month frequency, duration and modalities of transport in different contexts: at work (during work-time including outside of the main workplace), during commuting and for utilitarian purposes (e.g. errands, defined as non-commuting non-leisure purposes such as shopping, bringing children to school, going to the movies, etc.). Concerning non-transport-related SB, two sets of questions were asked. A first set of questions asked about time spent sitting in different contexts (at work, during transport and during leisure). A second set of questions were asked to obtain details about sedentary time spent in different leisure activities (total screen time, television/DVD, computer/video games/tablet, reading, sewing/knitting…). The English translation of the STAQ is available as additional file (See Additional file 1).

Cognitive testing of the STAQ was performed through face-to-face interviews in 32 subjects (18 men and 14 women aged 18 to 65 years with various occupations). Understanding and interpretation of the different questions were rated as fair to good with a global comprehension score of 26.4/28 for the items about transport and sedentary occupations.

### Population and protocol

A group of 103 healthy volunteers aged 20–65 years and living in the region of Lyon (France) were recruited by advertisement for the test-retest reliability and the validity study. Participants were selected as having no major physical disabilities, and to represent a large range of work- and leisure-related PA levels, as estimated by interview, with a sex ratio of about 50 %. All participants were equipped with a hip-worn accelerometer (Actigraph GT3X + ^TM^, ActiGraph Ltd, Pensacola, FL, US). They were instructed to wear the device when awake throughout the 7-day study period and asked to prospectively fill a contextualized activity logbook during the same period. For a subgroup 45 subjects (the first consecutive subjects of the total study group while maintaining a sex ratio of 50 %), the accelerometry and contextualized activity-logbook validation study was extended to 14 days, during which AEE was further measured using the reference DLW method. The STAQ was self-administered in all subjects at the end of the 7- to 14-day validation study period. Among the subjects with a 7-day validation accelerometry- and contextualized activity-logbook, 32 subjects (17 women, 15 men) aged 20–62 y, completed the STAQ a second time, one-month apart and in the same conditions, for the test-retest reliability assessment. The study was approved by the French Sud-Est 2 Institutional Review Board and all subjects provided written informed consent.

From the initial sample, 7 subjects were excluded because their questionnaires were not correctly or completely filled (*n* = 4) or because the study time period did not correspond to routine activities (e.g. holidays; *n* = 3). Data presented here were thus obtained from a final sample of 96 subjects (45 men and 51 women). Eight subjects, for whom logbooks were not reliable, were excluded from the analyses comparing the questionnaire outputs with logbook data. Valid accelerometry data (see below) were available for 88 subjects.

### Contextualized-activity logbook

Participants were asked to fill a pre-formatted contextualized activity-logbook throughout the 7-day or 14-day validation study period. More specifically, they were required to prospectively record time when awake and sleeping, as well as all the activities that they undertook throughout the day, by blocks of 30 min and by specifying context (home, workplace,…), posture (lying, sitting, standing, moving), as well as start time and duration (in min) of each activity bout. For transport, they were additionally required to specify each of TRA with their duration, place where it started and ended, and posture. Reported activities, postures and contexts were used to define different overall summary variables and context-specific activities corresponding to the questionnaire items. Participants with a minimum of 5 valid days, including one weekend day, were included for the validity analyses. Time spent in the different overall and context-specific activities was calculated for an average day (with a week corresponding to 5 week days and 2 week-end days).

### Accelerometry and sedentary time criterion measure

The Actigraph GT3X + ^TM^ worn at the hip during all the awaking time was used to objectively assess total and context-specific sedentary time. The Actilife version 6 software (Actigraph, Pensacola, FL, USA) was used to download and analyze data at the end of the validation period. The software normal filter was applied to the raw data and a 150 counts per min threshold excluding sleep-time was used to define sedentary time. A better accuracy for sitting time and SB estimation has been shown for this threshold rather than the use of a 100 counts per minutes threshold or of waist- or hip-worn inclinometry [16, 26]. Annotating the accelerometry-based sedentary time with the contextualized logbook data further enabled the calculation of objective context-specific sedentary times and allowed detailed validity assessment of the SB questionnaire in terms of context. Time spent in total and context-specific sedentary times were further calculated for an average day (with a week corresponding to 5 week days and 2 week-end days). Subjects who did not have at least 5 days including one week-end day were excluded from the analyses (n = 8). Data from 88 subjects were available for the total sitting time validity study and from 82 subjects (with both accelerometry and contextualized activity data) for the context-specific SB validity study.

### Activity energy expenditure (AEE) estimation and double-labelled water (DLW) reference measurement

Briefly AEE was estimated from the STAQ, as previously described for the RPAQ [24], by multiplying time participation in each activity item by its metabolic cost, and by adding an estimated AEE corresponding to time not accounted for by the questionnaire, which was arbitrary assigned a 0.2 MET value in individuals reporting their main transportation mode as active, and 0 otherwise.

AEE was measured with the DLW reference method as previously described [27, 28], using a 14-day DLW protocol for free-living total energy expenditure determination and indirect calorimetry (Quark RMR; Cosmed Medical systems corp US) for rest metabolic rate measurement.

### Statistical analysis

Descriptive data are presented as number (percentage) for categorical variables, and as means (± standard deviation [SD]) for continuous variables.

Test-retest reliability was assessed using single-measure intra-class-coefficients (ICC) and 95 % confidence interval (CI) with ICC <40 indicating poor agreement, 0.40–0.60 fair agreement, 0.60-0.80 good agreement and >0.80 excellent agreement.

Spearman rank order correlations and ICC with 95 % CI were used to examine the association between STAQ-derived items and AEE with corresponding 1) contextualized accelerometry-derived sedentary times for total sitting time and context-specific SB, 2) context-specific logbook data for total and specific TRA according to the different contexts, 3) DLW-measurement of AEE. Absolute biases were calculated as STAQ estimates minus their validity criterion measurements. Bland–Altman plots were used to further assess the degree of agreement between the STAQ estimates and the objectively measurements for total sitting time and AEE. Analyses were performed using SAS software 9.4 (SAS Institute Inc., Cary, NC, USA).

## Results

### Subjects characteristics

Characteristics of the 96 study subjects are shown on Table 1. Half of subjects were of male gender, and two thirds were employed (partial or full-time job) or students. There were no significant differences between employed and non-employed subjects in terms of activities, except for a higher time for active transport and lower total sitting time for the non-employed subjects (data not shown).

**Table 1:** Characteristics of the population (*n* = 96)

	N (%) or Mean ± SD ^a^
Gender, male	45 (47)
Occupational status, working	63 (66)
Overweight	35 (37)
Age (years)	40.5 ± 14.3
BMI (kg/m^2^)	27.1 ± 5.8
Sedentary behaviors	
Sitting time (h/wk) ^b^	
Total	46.5 ± 23.2
Work	15.6 ± 16.0
Transport	4.5 ± 4.0
Leisure time	26.4 ± 16.9
Leisure sedentary behaviors (h/wk) ^b^	
Total	42.8 ± 24.4
Total screen time	35.6 ± 22.1
Computer/tablet/video games	17.3 ± 13.1
TV/DVD	18.3 ± 13.1
Reading, writing, listening to music, sewing	7.3 ± 7.6
Transport-related activities	
Active transport (h/wk)^b^	
Walking	2.7 ± 3.9
Cycling	0.1 ± 0.3
Walking + cycling	2.8 ± 3.9
Passive transport (h/wk) ^b^	
All transportation type	6.8 ± 6.4
Car	4.4 ± 5.7

^a^unless otherwise specified (n (%)). ^b^As assessed by the STAQ

Time spent in passive transport was about twice the time spent in active transport. Total sitting time was on average 46.5 h per week, with more than 50 % spent as sitting during leisure time. Screen viewing represented more than 80 % of time spent in leisure SB (half in TV/DVD viewing, half in computer/video games). Characteristics of the subjects and the time they spent in the main activities did not differ between the sub-samples involved within the reliability and 7-day or 14-day validity studies.

### Test-retest reliability

Test-retest reliability results are presented in Table 2. The reliability was fair for total sitting time with an ICC of 0.52. Concerning context-specific SB and TRA, the highest ICC was noted for time spent viewing TV/DVD (ICC = 0.79). Good to excellent reliability (ICC > 0.60) was found for sitting time at work, total and specific (TV/DVD and computer/tablet/video games) leisure screen times, and transport-related walking and car use. Reliability was fair with ICC of 0.47-0.50 for total active and passive TRA and poor for total sitting time in leisure (ICC = 0.37) and transport (ICC = 0.28) contexts.

**Table 2:** Test-retest reliability data for sedentary behaviors and transport-related STAQ items (n = 32)

	Time spent (h/wk)		ICC [95 % CI]
STAQ items	Session 1 mean ± std	Session 2 (mean ± std)	
Sedentary behaviors			
Sitting time			
Total	45.3 ± 23.1	46.8 ± 20.3	0.52 [0.22;0.73]
Work (*n* = 13)	15.3 ± 13.9	18.5 ± 14.9	0.71 [0.49;0.84]
Transport	6. 3 ± 4.7	5.6 ± 4.5	0.28 [−0.06;0.56]
Leisure time	23.7 ± 13.5	22.7 ± 15.2	0.37 [0.03;0.62]
Leisure sedentary behaviors			
Total	34.8 ± 29.6	36.4 ± 21.4	0.64 [0.38;0.80]
Total screen time	27.1 ± 19.9	31.0 ± 19.8	0.70 [0.48;0.84]
TV/DVD	13.1 ± 10.7	15.4 ± 10.2	0.79 [0.61;0.89]
Computer/tablet/video games	14.0 ± 15.8	15.6 ± 15.0	0.64 [0.38;0.80]
Reading, writing, listening to music, sewing	7.8 ± 15.0	5.4 ± 5.5	0.26 [−0.08;0.55]
Transport-related activities			
Active transport			
Walking	2.6 ± 4.0	2.0 ± 3.5	0.61 [0.35;0.79]
Walking + cycling	2.8 ± 4.0	3.0 ± 5.6	0.47 [0.16;0.70]
Passive transport			
All transportation type	7.0 ± 5.2	6.9 ± 5.7	0.50 [0.20;0.72]
Car	5.0 ± 5.5	4.9 ± 5.7	0.67 [0.42;0.82]

### Validity

**Fig. 1:**
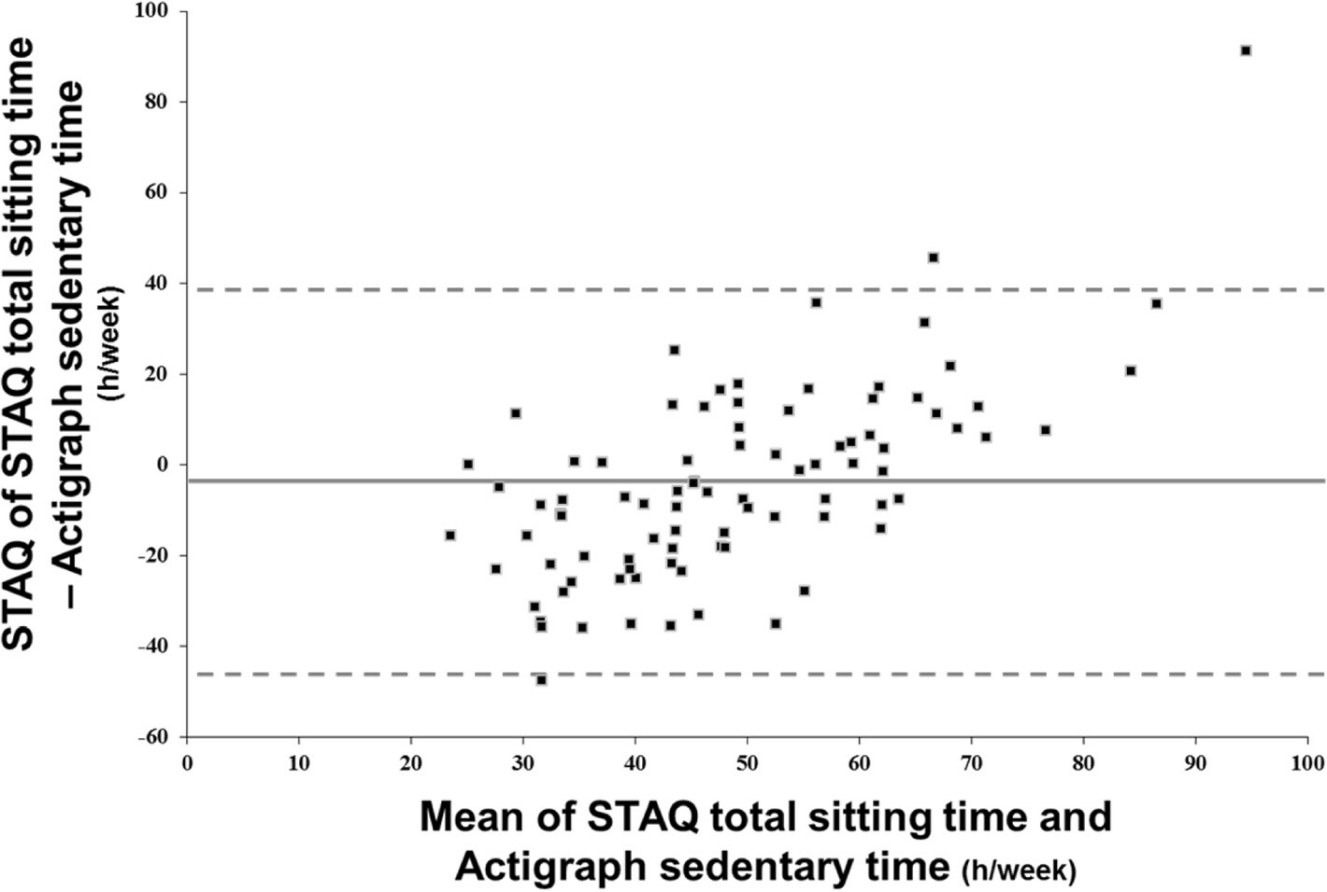
Bland and Altman visual analysis for total sedentary time as assessed by questionnaire (Sedentary, Transport and Activity Questionnaire, STAQ) and contextualized accelerometry (*n* = 88)

Validity analyses of the STAQ items addressing SB, as compared to contextualized accelerometry using logbook annotations, are presented in Table 3. A significant correlation and a fair to good agreement was found between the questionnaire estimate and the objective assessment for total sitting time (*r* = 0.54; ICC = 0.44) albeit with an important underestimation (−17 h/week) and wide limits of agreement (−43.8 to + 34.6 h/week) as illustrated by Bland-Altman plot (Fig. 1). Concerning context-specific items, the strongest association was found for sitting time at work (*r* = 0.88; ICC = 0.82) whereas low to moderate validity was found for time spent TV/DVD viewing (*r* = 0.46; r = 0.34) or in front of other screens during leisure time (*r* = 0.42; ICC =0.45), as well for total sitting time in transport (*r* = 0.35; ICC = 0.53) and leisure (*r* = 0.19; ICC =0.33) contexts. The questionnaire generally overestimated work- and leisure-related SB and sitting times, while it underestimated transport-related total sitting time. Compared to contextualized logbook, STAQ estimates of TRA was higher for car (*r* = 0.65) than for active transport (*r* = 0.41).

**Table 3:** Comparison of STAQ and contextualized accelerometry* estimates of time spent in different sedentary activities by contexts (*n* = 88 for total sitting time and *n* = 82 otherwise)

STAQ sedentary behaviours items	Biases ^a^ (h/wk)	Spearman correlation		ICC [95 % CI]
		r	p	
Sitting time				
Total	- 17.0 ± 18.1	0.54	<0.0001	0.44 [0.25 ; 0.60]
Work	+2.6 ± 8.6	0.88	<0.0001	0.82 [0.73 ; 0,88]
Transport	- 1.4 ± 3.5	0.35	0.001	0.53 [0.35 ; 0.67]
Leisure time	+17.6 ± 10.0	0.19	0.09	0.33 [0.12; 0.51]
Specific sedentary behaviours				
Total	+29.2 ± 19.4	0.33	0.002	0.18 [−0.04 ; 0.38]
TV/DVD	+7.3 ± 10.6	0.46	<0.0001	0.34 [0.14 ; 0.52]
Computer/tablet/video games	+8.4 ± 12.0	0.42	<0.0001	0.45 [0.26 ; 0.60]

^*^Accelerometry was contextualized using annotation from the logbook

^a^Calculated as STAQ estimate minus the objective accelerometry

Comparison of the estimates of time spent in TRA by the STAQ and the contextualized logbook are shown in Table 4. A significant and good correlation was noted for transport by car (*r* = 0.65; ICC = 0.66) although there was an underestimation of by about 50 % by the questionnaire. Lower associations were noted for active transport (r around 0.40; ICC = 0.38) with an overestimation of about 10 % with the questionnaire, as compared to the logbook.

**Table 4:** Comparison of time spent in transport-related activities as assessed by the STAQ and by the contextualized logbook (*n* = 88)

STAQ Transport related items	Biases ^a^ (h/wk)	Spearman correlation		ICC [95 % CI]
		r	p	
Active transport (h/wk)				
-Walking	+1.0 ± 3.6	0.39	<0.001	0.38 [0.19 ; 0.54]
-Walking + cycling	+0.9 ± 3.6	0.41	<0.001	0.38 [0.18 ; 0.54]
Passive transport				
-All transportation type	- 0.4 ± 5.3	0.36	0.001	0.40 [0.21 ; 0.56]
-Car	- 1.7 ± 3.6	0.65	<0.001	0.66 [0.52 ; 0.76]

^a^Bias was calculated as (STAQ-logbook) for time spent in transport-related activities

A significant correlation was found between the STAQ estimate of AEE and the DLW reference measure (*r* = 0.35). As illustrated by Bland-Altman plot (Additional file 2), the bias (7.86 (SD 3.81) kcal.kg^−1^.d^−1^ versus 8.22 (SD 5.07) kcal.kg^−1^.d^−1^was low (less than 5 %, *p* = 0.70) and, although relatively wide limits of agreement between both methods, no proportional error was found.

## Discussion

The present study provides evidence for the test-retest reliability and validity of the assessment of total and context-specific SB and TRA by the self-reported STAQ instrument in adults. The present results rely on criteria measures obtained from both contextualized logbooks and more robust objective measures of SB derived from hip-worn accelerometry. Reliability and validity were fair to good for total sitting time and more specifically work-related sitting time, for leisure screen-related SB and car use, but were lower for total sitting time in leisure and active transport-related activities. The questionnaire generally overestimated work- and leisure-related SB and sitting times, while it underestimated total and transport-related sitting times.

### Reliability

We found that the STAQ had moderate to good reliability, depending on SB or TRA items considered. Better reliability was noted for well-identified activities, both for SB and TRA. These activities may be easier to recall as they relate to specific and often time-limited activities (e.g. well-known journey or TV program). Reliability was lower for activities which are generally associated with less defined time frame (e.g. sitting time during transport, reading, writing, listening to music, sewing) or with less regular occurrence. For SB, reliability was comparable to other questionnaires used for assessment of TV viewing, screen time or sitting at work, sedentary time during leisure or transport [14, 29, 30]. For these 2 latter activities, reliability was slightly better with the Sedentary Behaviour Questionnnaire (SBQ) that focuses exclusively on SB [19]. For TRA, a recent paper reported similar reliability values for walking (transport or recreation ICC = 0.59-0.61, respectively) [10]. The authors observed that the reliability was higher for the frequency of using specific active transports than for the duration of transport use itself.

### Validity

The validity of the STAQ items for SB (total and in specific contexts) was evaluated using an objective measure of sedentary time based on both accelerometry and logbook, in order to contextualize accelerometry data. Accelerometry rather than inclinometry was used to objectively estimate sedentary time, as inclinometer has been found to provide less accurate estimation of sitting time when hip-mounted (as in our study) compared to thigh-mounted, with misclassification of about 30 % [16, 17]. Moreover, in these conditions, accelerometry with a threshold of 150 counts per minute outperformed the inclinometer Actigraph function [31]. The correlation coefficient (0.54) found between STAQ estimate and accelerometry for overall sitting time compares well with the range of correlation coefficients (r from 0.07 to 0.61) obtained with previous questionnaires [14, 32–34]. Similarly the validity of context-specific SB assessed by STAQ was in the range of the few other questionnaires that have used similar log-accelerometry combined validation criterion [2, 14, 17–19, 21] with r of 0.88 versus 0.25-0.63 for work; of 0.35 versus 0.07-0.46 for transport; of 0.19 versus 0.13-0.56 (not always including TV) for leisure time, of 0.46 versus 0.16-0.84 for TV time. The SIT-Q-7d, for which validation study was performed by positioning the accelerometer on the thigh of the participants displayed higher correlations except for sitting at work which correlation was higher with STAQ than with any other questionnaires [21]. Conversely with some but not all [35, 36] previous reports, total sitting time evaluated by STAQ was underestimated compared to the accelerometer data, whereas times spent sedentary at work, during leisure or watching TV were slightly overestimated in STAQ. It has been suggested that such dissection of behaviors may increase the accuracy of reporting compared to a global question [30, 35]. We can also hypothesize that it may be easier to recall sitting time spent in very specific contexts. Of note regarding biases for SB in specific contexts, STAQ questions were formulated to assess time spent in different activities and not specifically sitting while performing these activities. We cannot exclude that short standing breaks during sitting at work or watching TV have been included in the time reported in the STAQ and not recorded with the accelerometer, even if accelerometry rather than inclinometry was chosen as validation criteria which should to limit such discrepancies [15, 37].

As for assessment of TRA, time spent in active transport estimated by the STAQ did not differ significantly from those obtained by the logbook. However, time spent in passive transport tended to be underestimated with the questionnaire, as compared to the logbook. Our results also indicate that both tools provided similar estimates of time spent in structured activities (active transport or time spent sitting at work), while less structured activities such as sitting time (during leisure and transportation) were underestimated when assessed by the questionnaire as compared to the logbook even if displaying a fair concordance. Such discrepancies in over or underestimation have been reported previously according to activity type [14] or to individual differences in SB or PA levels [29]. This might be linked to difficulties for individuals to recall less structured activities or activities of very short length. It also has to be stressed that validation studies for different questionnaires vary according to the characteristics of the population (type, size, age), of the time frame (1, 7 or more days of observation), and of the logbook used.

To assess overall validity of STAQ, we finally compared AEE assessment from STAQ estimates with the DLW gold standard method. As for previous questionnaires [24, 38], correlation coefficient between the two estimates was less than 0.60. However, the bias was lower than reported with previous questionnaires, which may be due to the better insight in TRA and various specific SB leading to less time periods without behavior information, and for which no AEE estimation can be proposed.

### Strengths and limitations

One strength of the present study is the combined use of accelerometry with contextualized logbook to objectively assess the validity of overall and specific SB in several contexts. Presently, the STAQ asks about participation in a number of different types and contexts of SB and TRA to provide more specific behavior measures. Concerning the lack of a consensual gold standard method to assess validity of both PA and SB, as previously emphasized in the literature [6, 14], concurrently collecting behavior and context log data with accelerometer data appeared as the optimal way to validate activities in context, providing complementary critical aspects of activities [21]. There are some limitations that need to be mentioned. We did not perform an objective validation study of active transport times using GPS localization for example. We also acknowledge the potential bias coming from the weak representativeness of the study period of past 4 weeks as compared to the one-to-two week reference measures. Of note, no substantial difference was found between the 14-day protocol and the 7-day data.

## Conclusions

In summary, results from this study indicate that the STAQ has an acceptable test-retest reliability and a good ranking validity for specific questions regarding SB and TRA. More particularly for SB, we observed good overall validity of the STAQ for the context-specific assessment of total time spent sitting, more particularly for time spent sitting at work and for TV and leisure screen times. For time spent in transport-related activities, validity was moderate for active transport to good for car use. The STAQ appears thus as a useful easy-to-use tool for future studies that aim to concurrently assess specific SB and TRA, along with PA, in the context where they are performed in every-day life conditions.

This work will have to be considered within the perspective of new connected devices development and it is very likely that further improvement of movement components assessment will require both objective and self-reported tools to be optimally monitored.

## Abbreviations

AEE, activity-energy expenditure; CI, confidence interval; DLW, double-labeled water; ICC, intraclass correlation coefficient; METs, metabolic equivalents tasks; PA, physical activity; RMR, resting metabolic rate; RPAQ, recent physical activity questionnaire; SB, sedentary behavior; SBQ, sedentary behaviour questionnnaire; SD, standard deviation; STAQ, sedentary, transportation and activity questionnaire; TRA, transport-related activities

## Additional files

Additional file 1: STAQ, Sedentary, Transportation and Activity Questionnaire, English translation. Questionnaire. (PDF 433 kb) Additional file 2: Bland and Altman visual analysis for physical activity energy expenditure (AEE) as assessed by questionnaire (Sedentary, Transport and Activity Questionnaire, STAQ) and by the double-labelled water (DLW) method (*n* = 45). (DOCX 38 kb)
