# Supplementary material for: Assessment of sedentary behaviors and transport-related activities by questionnaire: a validation study
Source: BMC Public Health. 2016 Aug 9;16:753. doi: 10.1186/s12889-016-3412-3 (PMC4977835; doi:10.1186/s12889-016-3412-3)
Supplement: Additional file 1: — STAQ, Sedentary, Transportation and Activity Questionnaire, English translation. Questionnaire. (PDF 433 kb) [file 12889_2016_3412_MOESM1_ESM.pdf]

STAQ

The SEDENTARY, TRANSPORTATION AND ACTIVITY QUESTIONNAIRE

This questionnaire is designed to find out about your sedentary, transportation and physical activities in your everyday life in **the last 4 weeks**.

This questionnaire is divided into 5 sections:

- **Section A** is about your physical activity patterns in the house
- **Section B** is about your activity at work
- **Section C** is about your transportation activities
- **Section D** is about your leisure activities
- **Section E** is about your physical activities in general

Your answers will be treated as strictly confidential and will be used only for medical research.

Please answer every question, except when you are explicitly asked to ignore some of the questions.

What is your employment status?

☐ Full-time
☐ Part-time
☐ Student, training
☐ Retired or early retirement
☐ Disability
☐ Unemployment, income support,…
☐ Without employment (has never worked or has stopped working but does not correspond tone of the previous conditions)

What is your main activity ? ____

If you have a second regular activity, please precise: _______________________________

Section A : Home activities

In the last 4 weeks, how much time did you spend on average doing household activities from moderate to high intensity (such as vacuuming, wiping the floor or any comparable activities): |__||__| **hours** |__||__| **minutes per week**

Address of your principal residence/home:
Street : ____
Postcode : ______________________________
City : ____
State/Region: ____
Country : _________________________________

Section B : Activity at work / studying

Have you been in paid employment OR have you done regular voluntary work OR have you been studying/training at any time during the last 4 weeks?

☐ YES ☐ NO

If you have not been working (*paid employment, voluntary work, studying, training*) during the last 4 weeks, please proceed to "Section C: Transportation"

During 4 weeks, how many hours work did you do per week in total (***paid employment, voluntary work, studying, training***)?
Please indicate the number of hours per week without considering commuting time between residence and workplace.

4 weeks ago |__||__| H

3 weeks ago |__||__| H

2 weeks ago |__||__| H

1 week ago |__||__| H

Type of work

We would like to know **the type and amount of physical activity involved in your work**. Please tick (√) the option that best corresponds with your occupation(s) (*paid employment, voluntary work, studying, training*) ***in the last 4 weeks*** from the following four possibilities:

Please tick only one of the following

Sedentary occupation You spend most of your time sitting ☐

Standing occupation You spend most of your time standing or walking. However, your work does not require intense physical effort *(e.g. shop assistant, hairdresser, guard)* ☐

Manual work This involves some physical effort including handling of heavy objects and use of tools. *(e.g. plumber, electrician, carpenter)* ☐

Heavy manual work This implies very vigorous physical activity including handling of very heavy objects (*e.g. dock worker, miner, bricklayer, construction worker*) ☐

Please choose from 1 (*very sedentary work*) to 5 (*intense activity*) what best corresponds with the **intensity of physical activity demanded by your occupation** in the past 4 weeks. Please tick (√) one of the following options only:

1. Mainly sitting ☐
2. Combination of sitting/standing ☐
3. Mainly standing ☐
4. Some physical effort ☐
5. Heavy manual work ☐

Section C: Transportation

In the last 4 weeks, which mode of transport have you used **most often**, apart from transportation for recreation or leisure time?
Please tick (√) *one box only.*

Car/Motor vehicle ☐
Public transport ☐
Walk ☐
Cycle ☐
Other active transport (skate, rollers,…) ☐

If you have not been working (*paid employment, voluntary work, studying, training*) during the last 4 weeks, please proceed to "Section C.3: Utilitarian travels, out of work-related travels or travels for recreations and leisure"

Section C.1: Travel to and from work in the last 4 weeks

In order to evaluate your travels to and from your place of work, please indicate the address of your main place of work:

Address of your main place of work during the last 4 weeks:
Street : ____
Postcode : ______________________________
City : ____
State/Region: _______________________________________
Country : _________________________________

In the last 4 weeks, how many times a week did you travel from home to your main work? (*Count the outward journeys only*) |__||__|

We will now ask you to detail how many times and for how long you have used each form of transport in different conditions.

Please indicate in the following table **how many days per week**, ***during the last 4 weeks***, you have used each form of transport to go (and/or come back) from your work.

	**Number of days per week**
Car/Motor vehicle	|__|
Public transport	|__|
Walk	|__|
Cycle	|__|
Other active transport (skate, rollers,…	|__|

Please indicate in the following table **the average length of time per day**, ***during the last 4 weeks***, you have spent using each form of transport to go (and/or come back) from your work.

	**average time per day**
Car/Motor vehicle	|__||__| H |__||__| min
Public transport	|__||__| H |__||__| min
Walk	|__||__| H |__||__| min
Cycle	|__||__| H |__||__| min
Other active transport (skate, rollers,…	|__||__| H |__||__| min

Section C.2 Business and work-related travel out of your main work place in the last 4 weeks

Do you have to travel out of your main work place (i.e. client appointment, site visit,…), with the exception of home/work travels?

YES☐ ☐NO

If YES, please fill in the 2 following tables. **Please indicate in the first one how many days per week *during the last 4 weeks***, you have spent using each form of transport for your business travels (i.e. client appointment, site visit,…), with the exception of home/work travels.

	**Number of days per week**
Car/Motor vehicle	|__|
Public transport	|__|
Walk	|__|
Cycle	|__|
Other active transport (skate, rollers,…	|__|

Please indicate in the following table **the average length of time per day**, ***during the last 4 weeks***, you have spent using each form of transport for your business travels (i.e. client appointment, site visit,…), with the exception of home/work travels.

	**average time per day**
Car/Motor vehicle	|__||__| H |__||__| min
Public transport	|__||__| H |__||__| min
Walk	|__||__| H |__||__| min
Cycle	|__||__| H |__||__| min
Other active transport (skate, rollers,…	|__||__| H |__||__| min

<u>Section C.3: Utilitarian travels, out of work-related travels or travels for recreations and leisure, in the last 4 weeks</u>

Please indicate in the following table how many days per week *during the last 4 weeks*, you have spent using each form of transport for your utilitarian travels (i.e. shopping (grocery, post office, library, pharmacy,…), taking children to and from school, visiting relatives, going to the theatre,…), with the exception of work-related travels or travels for recreations and leisure
.

	**Number of days per week**
Car/Motor vehicle	|__|
Public transport	|__|
Walk	|__|
Cycle	|__|
Other active transport (skate, rollers,…	|__|

Please indicate in the following table **the average length of time per day**, ***during the last 4 weeks***, you have spent using each form of transport for your utilitarian travels (i.e. shopping (grocery, post office, library, pharmacy,…), taking children to and from school, visiting relatives, going to the theatre,…), with the exception of work-related travels or travels for recreations and leisure

	**average time per day**
Car/Motor vehicle	|__||__| H |__||__| min
Public transport	|__||__| H |__||__| min
Walk	|__||__| H |__||__| min
Cycle	|__||__| H |__||__| min
Other active transport (skate, rollers,…	|__||__| H |__||__| min

Section D : Recreation/Leisure

The following questions ask about how you spent your leisure time.

Please indicate in the following table how often you did each activity on average *over the last 4 weeks.*
Please indicate also the average length of time that you spent doing the activity on each occasion.

	**Number of times you did the activity in the last 4 weeks**							**Average time per episode**	
	None	*Once in the last 4 weeks*	*2 to 3 times in the last 4 weeks*	*Once a week*	*2 to 3 times a week*	*4 to 5 times a week*	*Every day*	*Hours*	*Minutes*
Walking for pleasure *(not as a means of transport)*									
Cycling for pleasure *(not as a means of transport)*									
Light gardening (watering, mowing the lawn, weeding or pruning)									
Heavy gardening (Digging, shovelling, chopping wood)									
DIY (carpentry, home or car maintenance)									
Backpacking, trekking									
Climbing									
Swimming leisurely									
Swimming competitive									
Jogging									
Floor exercises (stretching, bending)									
Relaxation exercices (yoga, Qi Qong)									
Conditioning exercises (using a bike or a rowing machine)									
Aquagym									
High impact aerobics or step aerobics									
Other types of aerobics (zumba…)									
Exercises with weights									
Racing or rough terrain cycling									

	Number of times you did the activity in the last 4 weeks							Average time per episode	
	None	*Once in the last 4 weeks*	*2 to 3 times in the last 4 weeks*	*Once a week*	*2 to 3 times a week*	*4 to 5 times a week*	*Every day*	*Hours*	*Minutes*
Competitive running									
Track and field									
Skiing, snowboard									
Tennis or badminton									
Squash									
Table tennis									
Bowling, indoor lawn or 10 pin									
Golf									
Football, rugby or hockey									
Rowing									
Volley-ball, netball or basketball									
Fishing or hunting									
Horse riding									
Snooker, billiards, darts									
Roller or ice skating									
Sailing, wind-surfing or boating									
Martial arts, boxing or wrestling									
Dancing (ballroom, disco)									
Musical instrument playing or singing									
Other activity 1 : ……………………..									
Other activity 2 : ……………………..									
Other activity 3 : ……………………..									

In the last 4 weeks, what is the average time you spent **per day** doing these activities **during your leisure time**, ***excluding working hours?***

	Working days												Non working days											
	0 to 15 min/day	15 to 30 min/day	30 to 60 min/day	1h to 2h/day	2h to 3h/day	3h to 4h/day	4h to 5h/day	5h to 6h/day	6h to 9h/day	9h to 12h/day	12h to 15h/day	> 15h/day	0 to 15 min/day	15 to 30 min/day	30 to 60 min/day	1h to 2h/day	2h to 3h/day	3h to 4h/day	4h to 5h/day	5h to 6h/day	6h to 9h/day	9h to 12h/day	12h to 15h/day	> 15h/day
Watching TV, DVDs or other videos																								
Using a computer, tablet, video games (non active)																								
Using active video games (WII®, Kinect®,…)																								
Sitting for reading, writing, knitting, sewing,…																								

Section E : Physical activity in general

Do you consider yourself as a physically active?
YES ☐ NO ☐

Are you involved in physical activity for health reasons?
YES ☐ NO ☐

Did you grow up in a family who valued physical activity and more particularly sport and exercise?
☐ YES, a lot
☐ YES, a little bit
☐ NO

Do you think that, for a healthy lifestyle, regular physical activity is….
☐ Very important
☐ Important
☐ Moderately important
☐ Of little importance
☐ Pointless
☐ No opinion

Time spent sitting

In the past 4 weeks, what is the average time you spent **per day** sitting in the different conditions:

	Working days												Non working days											
	0 to 15 min/day	15 to 30 min/day	30 to 60 min/day	1h to 2h/day	2h to 3h/day	3h to 4h/day	4h to 5h/day	5h to 6h/day	6h to 9h/day	9h to 12h/day	12h to 15h/day	> 15h/day	0 to 15 min/day	15 to 30 min/day	30 to 60 min/day	1h to 2h/day	2h to 3h/day	3h to 4h/day	4h to 5h/day	5h to 6h/day	6h to 9h/day	9h to 12h/day	12h to 15h/day	> 15h/day
During transport																								
During work													Non applicable											
During leisure time																								

Stair climbing

Please indicate how often you climbed up a flight of stairs (approx 10 steps) on average **per day *during the last 4 weeks.***
Please tick (✓) à on every line.

Number of times you climbed up a flight of stairs (approx 10 steps)	Average over the last 4 weeks					
	None	1 to 5 times a day	6 to 10 times a day	11 to 15 times a day	16 to 20 times a day	More than 20 times a day
On working days						
On non-working days						

Final question

Are the last 4 weeks representative of your regular physical activity ? YES ☐ NO ☐

Please detail ………………………………………………………………………………………………..
………
………
