# Supplementary material for: Assessment of sedentary behaviors and transport-related activities by questionnaire: a validation study
Source: BMC Public Health. 2016 Aug 9;16:753. doi: 10.1186/s12889-016-3412-3 (PMC4977835; doi:10.1186/s12889-016-3412-3)

**Additional File 2:** Bland and Altman visual analysis for physical activity energy expenditure (AEE) as assessed by questionnaire (Sedentary, Transport and Activity Questionnaire, STAQ) and by the double-labelled water (DLW) method (n=45).


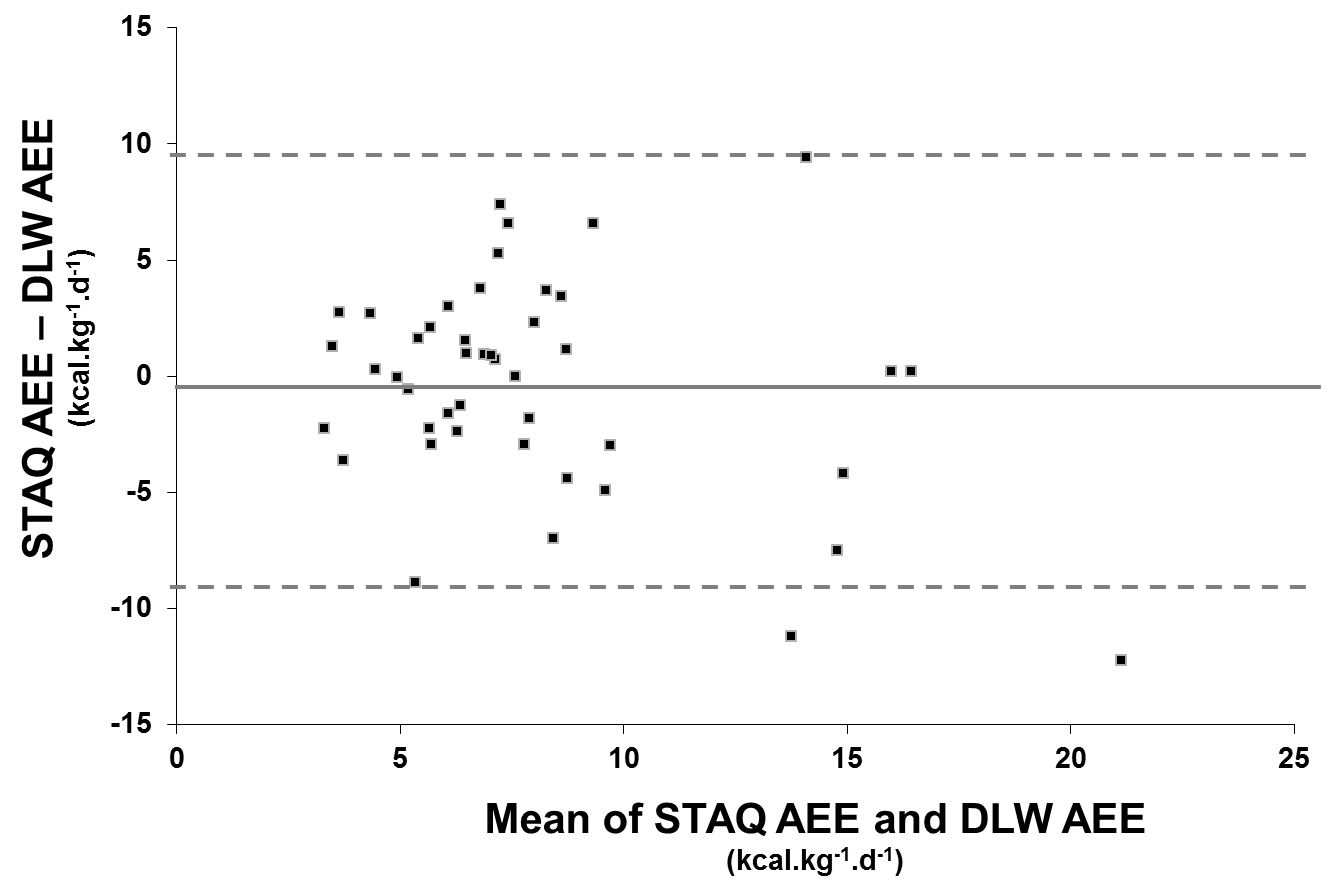

Supplement: Additional file 2: — Bland and Altman visual analysis for physical activity energy expenditure (AEE) as assessed by questionnaire (Sedentary, Transport and Activity Questionnaire, STAQ) and by the double-labelled water (DLW) method (n = 45). (DOCX 38 kb) [file 12889_2016_3412_MOESM2_ESM.docx]
